# The Prediction of Quality Parameters of Craft Beer with FT-MIR and Chemometrics

**DOI:** 10.3390/foods13081157

**Published:** 2024-04-11

**Authors:** Ofelia Gabriela Meza-Márquez, Andrés Ricardo Rodríguez-Híjar, Tzayhri Gallardo-Velázquez, Guillermo Osorio-Revilla, Oswaldo Arturo Ramos-Monroy

**Affiliations:** 1 Departamento de Ingeniería Bioquímica, Instituto Politécnico Nacional, Escuela Nacional de Ciencias Biológicas-Zacatenco, Av. Wilfrido Massieu S/N, Esq. Cda. Miguel Stampa, Col. Unidad Profesional Adolfo López Mateos, Zacatenco, Alcaldía Gustavo A. Madero, Ciudad de México C.P. 07738, Mexico; hijarandres4@gmail.com (A.R.R.-H.); gosorior@ipn.mx (G.O.-R.); oramosm@ipn.mx (O.A.R.-M.); 2 Departamento de Biofísica, Instituto Politécnico Nacional, Escuela Nacional de Ciencias Biológicas-Santo Tomás, Prolongación de Carpio y Plan de Ayala S/N, Col. Santo Tomás, Alcaldía Miguel Hidalgo, Ciudad de México C.P. 11340, Mexico; tgallardov@ipn.mx

**Keywords:** craft beer, quality parameters, quality control, FT-MIR spectroscopy, multivariate analysis

## Abstract

Beer is one of the oldest and most known alcoholic beverages whose organoleptic characteristics are the attributes that the consumer seeks, which is why it is essential to ensure proper quality control of the final product. Fourier transform mid-infrared (FT-MIR) spectroscopy coupled with multivariate analysis can be an alternative to traditional methods to predict quality parameters in craft beer. This study aims to develop prediction models based on FT-MIR spectroscopy to simultaneously quantify quality parameters (color, specific gravity, alcohol volume, bitterness, turbidity, pH, and total acidity) in craft beer. Additionally, principal component analysis (PCA) was applied, and it was possible to classify craft beer samples according to their style. Partial least squares (PLS1) developed the best predictive model by obtaining higher R^2^c (0.9999) values and lower standard error of calibration (SEC: 0.01–0.11) and standard error of prediction (SEP: 0.01–0.14) values in comparison to the models developed with the other algorithms. Specific gravity could not be predicted due to the low variability in the values. Validation and prediction with external samples confirmed the predictive capacity of the developed model. By making a comparison to traditional techniques, FT-MIR coupled with multivariate analysis has a higher advantage, since it is rapid (approximately 6 min), efficient, cheap, and eco-friendly because it does not require the use of solvents or reagents, representing an alternative to simultaneously analyzing quality parameters in craft beer.

## 1. Introduction

Beer is the most consumed alcoholic beverage in the world and the third most popular drink, after water and tea. Generally, it is elaborated with four main ingredients: starch-containing raw materials (generally malted barley), yeast, hop, and water [1]. Additionally, the liquid is saturated with carbon dioxide (CO_2_) or nitrogen gas (N_2_), which contributes to the foaming effect [2].

Beer has different nutrients such as carbohydrates (nonfermentable dextrins and α-glucans), proteins, B group hydrosoluble vitamins (folates, riboflavin, pantothenic acid, pyridoxine, thiamin, niacin), minerals (magnesium, potassium, sodium, calcium), and polyphenols [1].

Traditional beer companies dominate the global beer market; however, in recent years, the beer market has grown, and it now includes several hundred microbreweries that produce craft beers [3].

Craft breweries were first defined as “small independent manufacturers whose beer is unfiltered, unpasteurized, and without added nitrogen or carbon dioxide pressure during production”. Manufacturers emphasize their beers’ typical and distinctive flavor due to the addition of fruits, herbs, vegetables, and spices that can transform standard beer into specialty beer, along with other flavorings and fermentable substrates [4].

Subsequently, in the United States, the Brewers Association, through the term “craft”, distinguishes beer produced in small independent breweries from those beers produced in multinational corporations; in other words, craft beer is not elaborated by big corporations or macro breweries [5]. Therefore, the Brewers Association of the United States defines craft brewers as small brewers (annual production of 6 million barrels of beer or less) and independent (less than 25 percent of the craft brewery is owned or controlled by a member of the alcoholic beverage industry that is not itself a craft brewer) [6].

Craft manufacturing of beer depends on the intrinsic characteristics of the final product, for example, nutritional and sensorial peculiarities. In recent years, an increasing number of independent craft manufacturers have been focusing on using selected raw materials and soft production processes to obtain high-quality craft beer that can comply with the highest expectations of expert consumers. To many consumers, craft beer is considered a gourmet and select product, which is chosen because of the flavor diversity, and it is considered a higher quality product than traditional beer [4,5].

The rise in popularity of craft beer has led to the development of a higher number of craft beer styles, producing a new generation of products obtained in small breweries focused on producing craft beer styles, such as ales and lagers, and even some that cannot be classified in any of the main styles [4].

For the above, it is crucial to guarantee craft beer quality. Analyzing quality parameters in craft beer is essential to achieve efficient quality control and a prolonged useful life in the final product. The physicochemical parameters commonly analyzed in the beer industry for quality control in the final product are color, specific gravity, alcohol content, bitterness, turbidity, pH, and total acidity, among the most important ones [1,7].

To measure the physicochemical parameters previously mentioned, several processes or analytical methods are used, such as distillation, hydrometer, pycnometer, titration, ultraviolet-visible spectroscopy (UV-Vis), potentiometry, enzymatic assays, turbidimetry, nuclear magnetic resonance (NMR), capillary electrophoresis, refractometry, liquid chromatography (LC), and gas chromatography (GC), among others [7,8,9].

Previously mentioned analytic methods have shown their efficacy in determining physicochemical parameters in beer; however, some methods, such as LC and GC, are considered undesirable when the workflow is relatively rapid. Also, these techniques use large amounts of reagents and solvents (harmful for the analyst and the environment) and consume a lot of time because they require sample pretreatment for the analysis, which turns these methods inefficient because of the time/cost relation [10]. Also, it is estimated that less than 10% of craft breweries in the United States use sophisticated instrumental analysis to support their beer production. The above is due to the required instrumentation needing to be more affordable for small breweries, and it often requires specialized training and knowledge to operate correctly [1].

Nowadays, the tendency in food analysis demands the development of analytic techniques that are rapid, precise, reliable, cheap, simple, and environmentally friendly to replace the complex and expensive reference methods partially or entirely [8].

Fourier transform mid-infrared (FT-MIR) has proven to help analyze quality parameters in a wide variety of food [10]. This technique was proposed as an excellent alternative to conventional methods because when it is compared with GC and HPLC (high-performance liquid chromatography), it has several advantages, such as being rapid and non-destructive; moreover, it does not require sample preparation, it does not generate hazardous wastes; and it does not use solvents and reagents. It is also highly specific and sensitive [11]. Also, FT-MIR spectroscopy coupled with multivariate analysis has successfully developed prediction models that enable the rapid and simultaneous identification of qualitative and quantitative composition in various food matrices [12,13,14,15].

FT-MIR spectroscopy and multivariate analysis have been used to determine some quality parameters in craft beer. Duarte et al. [16] classified styles of beer (Ale, Lager, alcohol-free). Iñón et al. [17] and Llario et al. [18] analyzed alcohol content, original extract, and real extract. Lachenmeier [7] determined relative density, alcohol content, original gravity, pH, acidity, bitterness, and color. Polshin et al. [8] evaluated original, apparent, and real extract; alcohol volume; bitterness; and real and apparent degrees of fermentation. Finally, Castritius et al. [19] determined the limit of attenuation.

FT-MIR spectroscopy coupled with multivariate analysis has only been used to identify the authenticity of an Italian craft beer [20]. To our knowledge, no studies determine quality parameters simultaneously in craft beer through FT-MIR spectroscopy coupled with multivariate analysis. The objective of this study was to develop chemometric models based on FT-MIR spectroscopy to simultaneously quantify quality parameters (color, specific gravity, alcohol content, bitterness, turbidity, pH, and total acidity) in different craft beer styles to develop a rapid alternative methodology for the routine quality control.

## 2. Materials and Methods

### 2.1. Reagents

All reagents used for the chemical analysis were of analytical grade and purchased from JT Baker (Baker-Mallinckrodt, Ciudad de México, México).

### 2.2. Samples

A total of 60 samples from different brands that include 24 styles of craft beer from different manufacturers were donated by craft brewers or were bought in authorized and certified liquor stores in Mexico City, Mexico.

The style of craft beer corresponds to the established by the Beer Judge Certification Program [21]. The number of samples by the style of craft beer was the following: American Pale Ale (*n* = 8), Barleywine (*n* = 1), Belgian Blond Ale (*n* = 1), Berliner Weisse (*n* = 1), Black India Pale Ale (*n* = 1), Blonde Ale (*n* = 3), Brown Ale (*n* = 5), California Common (*n* = 1), Cream Ale (*n* = 2), Dark Lager (*n* = 1), Dubbel (*n* = 1), Golden Ale (*n* = 1), Imperial Stout (*n* = 2), India Pale Ale (*n* = 8), Kölsch (*n* = 1), Lager (*n* = 5), Oatmeal Stout (*n* = 2), Porter (*n* = 7), Scottish Ale (*n* = 1), Stout (*n* = 2), Sweet Stout (*n* = 2), Vienna (*n* = 1), Weissbier (*n* = 1), and Witbier (*n* = 2).

The number and selection of samples were made to cover a wide range of different quality parameters to maximize the variance and challenge any prediction model to obtain robust calibration models that could be applied to unknown craft beer samples.

### 2.3. Sample Preparation

All craft beer samples were stored in the dark at 4 °C until analysis. Before analysis, the craft beer samples were degassed in an ultrasonic bath (Ultracleaner, CScientific, CS-UB100, London, UK) at 5 °C to eliminate CO_2_.

### 2.4. Chemical Analysis

Chemical analyses were performed according to the Official Methods of the European Brewery Convention (EBC Analytica) and the Association of Official Analytical Chemists (AOAC). All chemical analyses were performed in triplicate.

The color was determined according to method 9.6 of EBC Analytica [22] using a spectrophotometer (Jenway, model 7305, Stone, Preston, UK), absorbance was measured at 430 nm, and the results were expressed as color units EBC (EBC = 25 × dilution factor × Abs_430nm_). Specific gravity was determined according to method 9.43.1 of EBC Analytica [23] using a pycnometer (Exelo 25 cm^3^). The alcohol content was determined according to method 9.2.3 of EBC Analytica [24] using an Abbe refractometer (Bellingham + Stanley Abbe 60), and results were expressed as alcohol percentage (% alcohol, *v*/*v*). Bitterness was determined in accordance with method 9.8 of EBC Analytica [25] using a spectrophotometer (Jenway, model 7305, Stone, UK), absorbance was measured at 275 nm, and results were expressed as international bitterness units (IBUs = 50 × Abs_275nm_). Turbidity was determined according to method 9.29 of EBC Analytica [26] using a turbidimeter (Hach, Mississauga, ON, Canada, 43900), and results were expressed as nephelometric turbidity units (NTUs). pH analysis was carried out according to method 9.35 of EBC Analytica [27] using a pH/conductivity meter (Oakton PC 700). Acidity analyses were performed in accordance with the AOAC Official Method 950.07 for beer [28]; results were expressed as mg lactic acid/L.

### 2.5. Statistical Analyses

The results from the seven physicochemical parameters were analyzed using one-way analysis of variance (ANOVA) and Tukey’s multiple comparisons of means (*p* ≤ 0.05). Statistical analyses were performed with Minitab software version 17.1.0 (State College, PA, USA). Data were expressed as mean ± standard deviation.

### 2.6. FT-MIR Spectra

Before obtaining the FT-MIR spectra, craft beer samples were placed in an ultrasonic bath (Ultracleaner, CScientific, CS-UB100, London, UK) for 15 min to eliminate the interference from the dissolved carbon dioxide. Subsequently, samples were filtered with the Whatman^®^ paper filter (0.45 µm) to avoid the influence of insoluble particles.

FT-MIR spectra of each craft beer were collected with an FT-MIR spectrophotometer (model GX, PerkinElmer^®^, Hopkinton, MA, USA) that had a triglycine sulfate (DTGS) detector and included an attenuated total reflectance (ATR) accessory with zinc selenide (ZnSe) crystal (10 reflections, angle of 45°) (model 022-12xx, PerkinElmer Inc., Hopkinton, MA, USA). The spectra were recorded at constant humidity (30%) and controlled temperature (22 ± 1 °C) in the mid-infrared region (4000–550 cm^−1^), with 64 scans, resolution of 4 cm^−1^, and in absorbance (A) units. Before obtaining the FT-MIR spectra, an air background spectrum was registered under the same instrumental conditions of the sample. To obtain the FT-MIR spectra, approximately 1.0 mL of each degassed sample was placed on the ZnSe crystal, completely covering it and avoiding bubble formation. Subsequently, the ZnSe crystal was covered with a lid to avoid sample evaporation. After concluding each measurement, the ZnSe crystal was rinsed with distilled water and dried carefully with disposable tissue. The results were recorded in triplicate, averaged, and processed employing the software Spectrum version 6.3.1 (PerkinElmer^®^, Hopkinton, MA, USA).

### 2.7. Multivariate Analysis

#### 2.7.1. Principal Component Analysis (PCA)

PCA was performed to know the exploratory analysis of spectra and subsequently develop models to simultaneously predict quality parameters in craft beer. PCA was performed using the statistical software Minitab version 17.1.0 (State College, PA, USA).

#### 2.7.2. Chemometric Models

To select the sets used to calibrate and validate chemometric models, a population of heterogeneous samples was selected; this way, the prediction range (application) of the developed model would increase.

- Calibration

Multivariate analysis was developed with the software Spectrum Quant+ version 4.51.02 (PerkinElmer^®^, Hopkinton, MA, USA). Chemometric models to simultaneously predict quality parameters (color, specific gravity, alcohol content, bitterness, turbidity, pH, and total acidity) in craft beer were developed with principal components regression (PCR) and partial least squares (PLS1 and PLS2). These algorithms mathematically relate the absorbance of the FT-MIR spectra of each craft beer sample with the analytical values from each physicochemical parameter.

Samples were randomly separated into sets of calibration and validation. According to Polshin et al. [8] and Tamaki and Mazza [29], the calibration set must comprise 75% of the samples, and the validation set must comprise 25% of the samples. The calibration set consisted of 45 average FT-MIR spectra (75% of the samples).

For the data pretreatments, several mathematical tools were analyzed, as follows: the absorption band around 2382 and 2314 cm^−1^ (which is due to atmospheric CO_2_, was removed), Savitzky–Golay filter (13 smoothing points), Savitzky–Golay derivatization (first order), baseline correction (offset), and normalization (standard normal variates, SNVs).

The best model was selected by analyzing the following statistical data: (i) optimal number of factors: corresponds to the minimum value in the standard error of prediction (SEP); (ii) R^2^c: coefficient of determination, which indicates the correlation of the real value against the estimated value by the chemometric model. R^2^c must be close to 1; and (iii) standard error of calibration (SEC), which indicates if the model adjusts or not to the calibration data. SEC must be as low as possible.

Given that the capacity of the model to adjust to calibration data is not a direct measurement of the prediction capacities, it is mandatory to compare predicted values for new samples that were not used to build the model. Hence, a validation of the developed models was made.

- Validation

Chemometric models were validated with 15 average FT-MIR spectra (25% of the samples) [8,29]. Samples from the validation set were used to validate the calibration model and ensure that a stable model was achieved. To verify and validate the results, the following statistical data were analyzed: (i) R^2^v: coefficient of determination of validation, which indicates the error in the regression for the predicted values. R^2^v must be as close as possible to 1. (ii) SEP: standard error of prediction, which evaluates the precision with which the samples of prediction are adjusted to regression. SEP must be as low as possible. (iii) MD: Mahalanobis distance, which must be lower than 1. (iv) RR: residual ratio, which must be lower than 3. (v) %RD: relative difference percentage, which must be lower than 10%. (vi) RPD: residual predicted deviation, which evaluates the prediction error in terms of standard deviation. RPD must be as high as possible [8,30,31].

- Prediction (application)

The model was applied to ten samples of craft beer not included at the calibration and validation stages. Samples from the prediction set were used to evaluate the prediction ability of the developed model. The application of the model is essential to evaluate the prediction of unknown samples. Generally, the obtained results are acceptable. Hence, this stage is a second evaluation of the prediction ability of the model [32].

## 3. Results

### 3.1. Chemical Analysis

The physicochemical parameters of craft beer are presented in Tables S1–S6. There is a statistical difference (*p* ≤ 0.05) in all chemical analyses, even in the samples from the same beer style. These differences may be due to the use of different raw materials and different production processes at each craft brewery.

The results of color of craft beer from different styles (Table S1) varied significantly (*p* ≤ 0.05) from 2.1 units EBC (Berliner Weisse) to 41.7 units EBC (Stout). Color values obtained complied with the accepted range (1–65 units EBC) by the Beer Judge Certification Program [21] and according to the literature on craft beer [33]. Differences of the color units EBC of the samples of the same style of craft beer can be due to problems in mashing, such as incorrect mashing time, bad grain milling, or miscalculation of the types of malt used in the recipe [34].

Color is a critical quality parameter since, generally, it is the first characteristic the consumer notices in a beer. Color in craft beer varies from pale yellow (wheat beer) with low color units EBC to opaque black (Imperial Stout) with high color units EBC. Color depends mainly on the toasting of malted barley and the types of malt and other grains (oat, wheat, unmalted barley, rice) added to beer [35].

The results of specific gravity of craft beer from different styles (Table S2) varied from 1.004 (Lager) to 1.020 (Oatmeal Stout). Specific gravity values were within the acceptable range (1.001–1.040) by the Beer Judge Certification Program [21]. Differences of specific gravity values of samples of the same style of craft beer were probably due to mashing and fermentation, which cause the lack or excess of specific gravity in beer [36]. For example, incorrect mashing time, bad grain milling, or miscalculation of the types of malt used in the recipe leads to enzymes that act in this process to be subjected to suboptimal conditions, which translates into a poor carbohydrate extraction from malt [34].

Fermentation is the process through which yeast turns sugars into alcohol and CO_2_ in any variations of fermentation times or temperatures, and the incorrect use of the variety of yeast can lead to an incorrect process of fermentation [37]. During the beer fermentation process, specific gravity decreases regarding the conversion of sugars in alcohol, so this process is measured with specific gravity since, in carbohydrates, specific gravity is higher than in water, and the specific gravity of alcohol is lower than in water [38]. Fermentation can be controlled using the specific gravity value. Therefore, values that do not fit brewery specifications are incorrect fermentations [34].

The results of alcohol content of craft beer of different styles (Table S3) varied from 4.0% *v*/*v* (Berliner Weisse) to 10.4% *v*/*v* (Barleywine). Alcohol percentages of craft beer were within those reported in the literature for craft beer [2,33] and within the established values (>2% <20% de alcohol, *v*/*v*) by the NOM-199-SCFI-2017 [39]. Differences in alcohol percentages of samples of the same style of craft beers are due to the same problems indicated in the determination of specific gravity, that is, incorrect mashing or fermentation since the alcohol volume depends on the proper fermentation process and carbohydrate extraction of malt [34]. The alcohol percentage is a decisive parameter in the quality of the beer, since the amount of it will determine, to a great extent, the taste of beer [40].

Bitterness values of craft beer of different styles (Table S4) varied from 9.0 IBU (Lager) to 72 IBU (Imperial Stout). Results for bitterness were within the accepted range (1–120 IBU) of the Beer Judge Certification Program [21] and according to the literature for craft beer [2,33]. Differences of international bitterness units of samples of the same style of craft beer can be due to the brewing process, specifically in the quantity of hop and with regard to moment of its incorporation. For example, while the wort is cooking and being mixed with hop, α-acids are isomerized to soluble iso α-acids that provide bitterness to beer [36]; therefore, if the brewmaster does not add hops in the proper amounts and at the correct times, bitterness will not be as desired [41].

Additionally, after packaging, beer starts losing its bitterness and gains sweet and caramel flavors; this change increases as time passes [9]. This is due to α-acids in the beer wort being found in the mix with cis and trans isomers, with a proportion of 68:32 parts favoring cis compounds, which have an average life span of five years. Meanwhile, trans compounds have an average life span of less than one year [42], which can be the main factor in obtaining different bitterness values than desired.

Bitterness of craft beer is a critical quality factor since it considerably influences flavor [36]. The bitterness value varies depending on the style; the higher the IBU index, the more bitter the product will be [43].

Turbidity results of craft beer of different styles (Table S5) varied from 2.0 NTU (Lager) to 201.3 NTU (Imperial Stout); these results coincide with those reported in the literature [44,45]. Clarity or lack of turbidity is one of the main objectives in some beer styles. Clarity is significantly related to the beer style. Generally, turbidity is undesirable in most styles; however, in Weissbier and Witbier beers, turbidity is desirable. Likewise, dark beers such as Brown Ale, Porter, or Stout accept certain turbidity levels [9].

Turbidity is an important parameter in beer production since it affects the product’s final quality. Beer has several ingredients, such as proteins, carbohydrates, polyphenols, fatty acids, and amino acids, among other compounds that can precipitate and form turbidity [46].

The pH values of craft beer of different styles (Table S6) varied from 3.35 (Witbier) to 5.03 (Oatmeal Stout); these results coincide with those reported in the literature [1,7,16,47,48]. The pH value in craft beer is an important quality parameter since it is fundamental in all beer production processes, from enzymatic reactions to microbiological processes [36].

Finally, acidity values of craft beer of different styles (Table S6) varied from 4.92 mg lactic acid/L (Blonde Ale) to 47.76 mg lactic acid/L (Oatmeal Stout); these results are found within the accepted range (≤10,000 mg lactic acid/L) of the NOM-199-SCFI-2007 [39]. The amount of lactic acid present in craft beer is an important parameter since it is produced by bacterial deterioration and can be used as a hygiene indicator from the production plant. High lactic acid concentrations suggest the presence of bacteria such as *Lactobacillus* or *Pediococcus* [48].

Results from the physicochemical parameters in craft beer presented a wide range of values, and this guarantees that the calibration set used to predict such physicochemical parameters includes the minimum and maximum values, since the precision and solidity of the prediction model are determined for the variability of the values used in the calibration set [8].

### 3.2. Spectra FT-MIR

**Figure 1:**
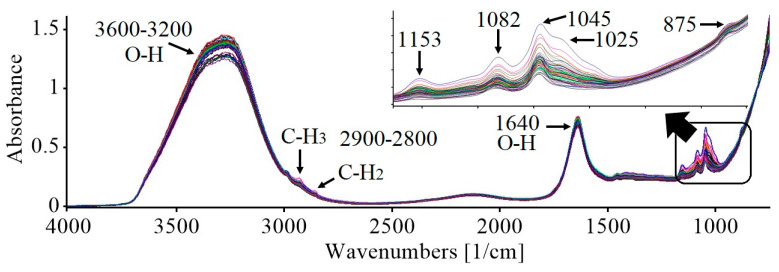
FT-MIR spectra of craft beer.

Figure 1 presents the FT-MIR spectra from the 60 craft beer samples in the mid-infrared region (4000–800 cm^−1^). The FT-MIR spectra reflect the chemical composition of craft beers (mainly water, alcohol, and carbohydrates), and even though beer is a food matrix with certain complexity, it is possible to define characteristic bands from functional groups present in molecules.

The band at 3600–3200 cm^−1^ is attributed to stretching vibrations of the O-H group corresponding to water and ethanol present in the samples [19,20]. The region at 2900–2800 cm^−1^ corresponds to the aliphatic vibrations due to the stretching vibrations of methyl and methylene group from the ethanol [8,18].

The atmospheric band of carbon dioxide (CO_2_) in the region between 2382 and 2314 cm^−1^ was not considered for the construction of the prediction’s models nor for the interpretation of the FT-MIR spectra. At 1640 cm^−1^, stretching vibrations of the functional group H-O-H are present [8,19].

The region of 1200–800 cm^−1^ corresponds to the fingerprint zone, and according to Polshin et al. [8] and Gordon et al. [2], absorption bands are difficult to assign to specific molecular vibrations due to the superposition of peaks associated with maltotriose-maltose and dextrins during beer production. Likewise, the peaks registered in the fingerprint zone can also be associated with the presence of ethanol in beer. Even though the fingerprint region is considered complex, the bands presented are mainly associated with vibrations of the C-C-O, C-O, and O-H bonds that correspond to carbohydrates (maltose, maltotriose, fructose, dextrins, and diverse oligosaccharides) and ethanol [8,18,20,48].

Bands at 1153 cm^−1^ and 1082 cm^−1^ correspond to maltose, which was expected, since it is the carbohydrate with the highest proportion in the wort. The band at 1045 cm^−1^ corresponds to maltotriose, and the little shoulder identified at 1025 cm^−1^ is assigned to dextrins. Likewise, peaks at 1082 cm^−1^ and 1045 cm^−1^ correspond to the stretching vibrations for the C-O bonds from primary and secondary alcohols. The peak at 875 cm^−1^ is due to the stretching vibration of the C–C–O bonds of ethanol [20,48]. The peak at 875 cm^−1^ may also be related to the C-H groups derived from esters and aromatic groups, as well as N-H from amine groups. The groups mentioned are generally related to beer style (Ale or Lager) [2].

As seen in Figure 1, the fingerprint area presented bands and peaks with a significant variation among craft beer samples; thus, generally, this region is used to characterize beer samples according to their alcohol content (low or high alcohol content) to classify the beer style (Ale or Lager) or the elaboration process (commercial or craft).

### 3.3. Multivariate Analysis

#### 3.3.1. Principal Components Analysis (PCA)

**Figure 2:**
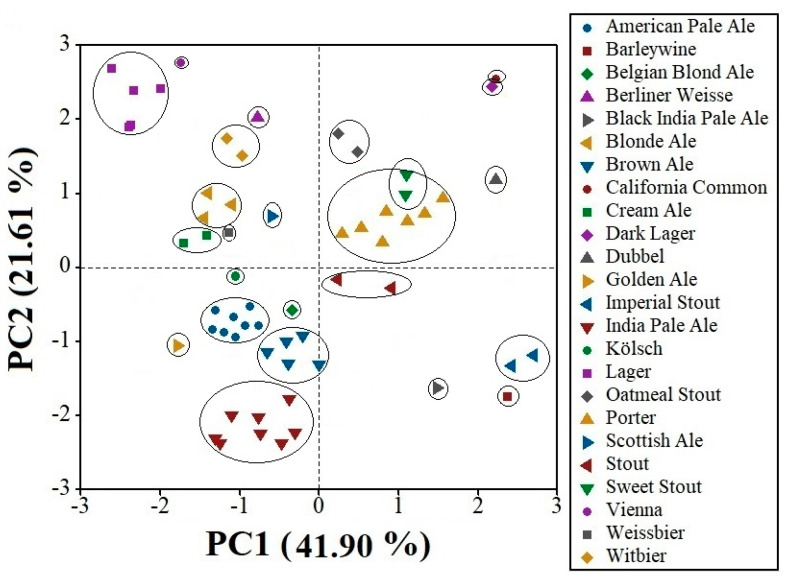
Score plot of beer samples analyzed with FT-MIR spectroscopy.

PCA was used to identify the exploratory spectral analysis of the craft beer samples. Figure 2 presents the score plot of the first two principal components (PCs) that explain 63.51% of the variability from the analyzed craft beer samples. The most considerable variability is in the first component (41.90%). The 60 craft beer samples were grouped in defined sets based on their beer styles (24 styles); this grouping is mainly related to the composition of the samples, which is due to the differences in the sugar and alcohol content present in each craft beer, and this was reflected in the FT-MIR spectra previously described.

#### 3.3.2. Chemometrics Models

To construct the models, the following spectral regions were used—3600–2800 cm^−1^ and 1700–850 cm^−1^—since this region presented the highest correlation of the absorbance of each wavelength with reference values from the physicochemical parameters. The spectral regions between 2382–2314 cm^−1^ and 840–550 cm^−1^ were eliminated as they are attributed to atmospheric CO_2_ and spectral noise, respectively.

Table 1 presents calibration data from the chemometric models for predicting the quality parameters of craft beer.

It was not possible to model the specific gravity parameter; this is because the range of reference values was minimal (1.005 to 1.030) and because the multivariate statistic program (Spectrum Quant+) was not capable of calibrating such parameter, showing a banner alert that indicated “zero variance”. Also, an individual model was built to predict specific gravity. However, it was not possible to construct the model due to the low variability of the reference values (1.005 to 1.030). Due to the above, this parameter was eliminated from the construction of the predictive models.

The calibration results from the chemometric models that were developed to predict quality parameters in craft beer indicate that the algorithm with the best predictive capacity was PLS1, which presented higher R^2^c (0.9999) values and lower SEC (0.01–0.11) and SEP (0.01–0.14) values in comparison to the models developed with PCR and PLS2 algorithms. PLS1 calibrates properties one by one, and PLS2 calibrates properties simultaneously. In contrast to PLS, PCR uses the properties of decomposing in principal components, performing an inverse multiple regression of the property to be determined on the scores (which reveal differences or similarities among samples) instead of performing it on the original data. This explains why PLS provides better results than PCR [30].

**Figure 3:**
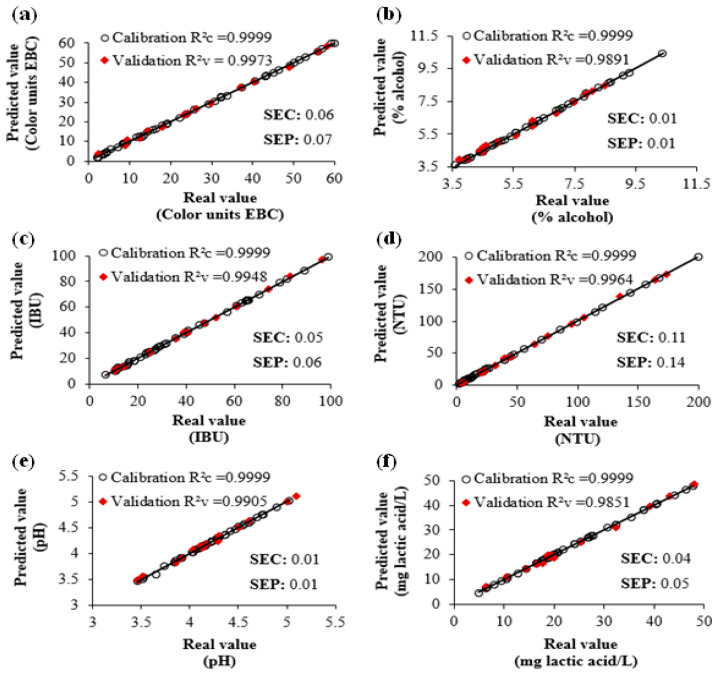
Plots of predicted values versus real values of (**a**) color, (**b**) alcohol, (**c**) bitterness, (**d**) turbidity, (**e**) pH, and (**f**) acidity for the calibration and validation samples determined using the PLS1 algorithm.

R^2^c values obtained in physicochemical parameters (except for specific gravity) indicate an excellent correlation between the reference values and predicted values by PLS1 (Table 1, Figure 3). Different authors point out that R^2^c values above 0.90 indicate “excellent precision”, and predictions are highly reliable [29,30]. Likewise, SEC and SEP values evaluate the precision with which calibration and prediction samples, respectively, fit to regression; thus, low values, such as the ones obtained with the model developed with PLS1, correspond to a better adjustment in data [30].

The number of factors corresponds to the minimum value of SEP. Therefore, the selection of the number of factors is decisive in the calibration process, since the number of factors allows us to know when the developed models are overfitting or underfitting. Beebe et al. [30] indicate that the number of factors must be under 50% of the samples used during calibration. The model developed with PLS1 accomplishes the previously established, since PLS1 used 10 factors in all the physicochemical parameters, representing 22.22% of the samples used during calibration. The above indicates that the model developed has great probabilities to provide adequate prediction results.

Validation results (Table 1, Figure 3) indicate that, for 15 external validation samples, the real values of the 6 physicochemical parameters match closely with the predicted values by the PLS1 model (R^2^v = 0.9851–0.9973). Therefore, the developed model presents good predictions for simultaneously determining quality parameters in craft beer. Likewise, to corroborate the reliability of the PLS1 model, a one-way ANOVA analysis was performed through a Dunnett’s test by comparing the real values with the predicted values for the six physicochemical parameters. The previous analysis demonstrated that between the real values and the predicted values with the PLS1 model, there is no significant difference (α = 0.05), demonstrating precision in the developed model’s predictions (Table S7).

The statistical parameters included in Table 1 were also considered to evaluate the predictive capacity of the PLS1 model. Results comply with the established limits (MD ≤ 1; RR ≤ 3; % RD ≤ 10%; RPD must be as high as possible) for all validation samples. The above indicates that the developed model with the PLS1 algorithm is useful for simultaneously predicting quality parameters (color, alcohol volume, bitterness, turbidity, pH, and total acidity) in craft beer accurately and precisely.

The quantification limits of the model are the following: color = 2.1 units EBC to 41.7 units EBC, alcohol volume = 4.0% *v*/*v* to 10.4% *v*/*v*, bitterness = 9.0 IBU to 72 IBU, turbidity = 2.0 NTU to 201.3 NTU, pH = 3.35 to 5.03, and total acidity = 4.92 mg lactic acid/L to 47.76 mg lactic acid/L).

**Figure 4:**
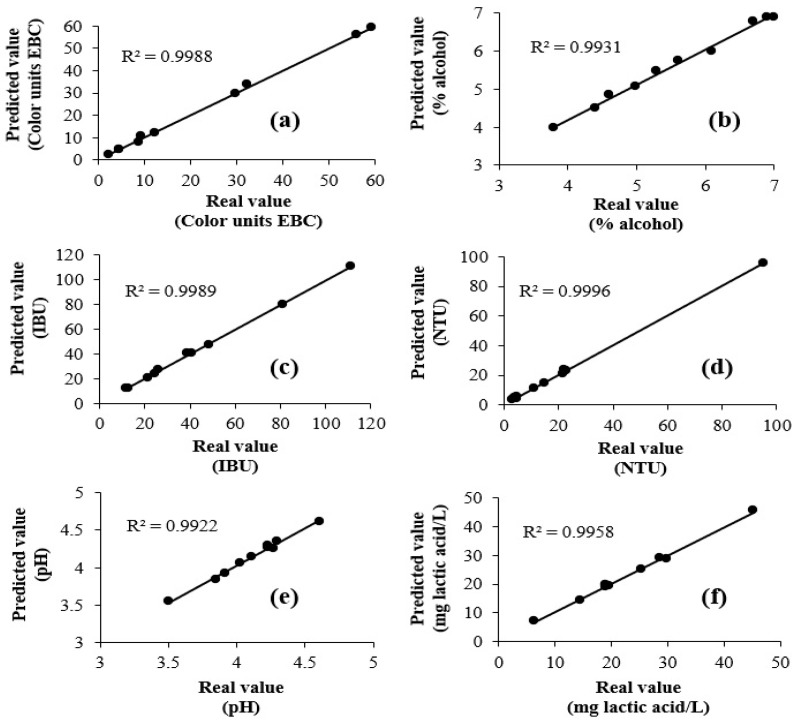
Plots of predicted values versus real values of (**a**) color, (**b**) alcohol, (**c**) bitterness, (**d**) turbidity, (**e**) pH, and (**f**) acidity for the samples used to apply the model developed with PLS1.

Once validated, the PLS1 model was applied to ten craft beer samples different from those used in the calibration and validation set. The accuracy of predictions is presented in Figure 4, where it is possible to observe that, for all physical parameters, predicted values with the PLS1 model are very close to those obtained with conventional chemical analysis (R^2^ = 0.9931–0.9996). The above confirmed that FT-MIR spectroscopy coupled to chemometrics provides similar results to the ones achieved with conventional analysis, but ones that are faster and cheaper to obtain, since it does not use reagents, solvents, and pretreatment of samples.

Finally, the model was applied to fifteen samples of non-craft beer (Table 2). The results showed that the model is capable of correctly predicting quality parameters (color, alcohol volume, bitterness, pH, total acidity, and turbidity) in non-craft beer.

## 4. Conclusions

The multivariate analysis applied to craft beer was very useful since PCA grouped 60 craft beer samples based on their beer styles. On the other hand, the model developed with PLS1 was accurate and reliable for simultaneously predicting the quality parameters of craft beer. Validation and prediction stages corroborated the obtained results. However, specific gravity could not be calibrated due to the low variability in the reference values. The alternative methodology developed in this study would be of great interest for small-scale breweries with low investment capabilities, but which require a great need to control processes, since small craft breweries work with highly variable and non-standardized processes compared to industrial breweries. FT-MIR spectroscopy coupled with chemometrics could be a simple and rapid screening tool for simultaneously quantifying quality parameters in craft beer. This study could be used as the basis for carrying out research related to the establishment of a rapid and accurate analytical method for determining the quality of craft beer. Further work should be carried out in commercial samples from craft beer to confirm the applicability of the developed models.

It is recommended to develop further predictive models that include other parameters relevant to quality control (metals, microbiological counts), health-benefiting attributes (polyphenols, flavonoids), and chemical composition. The above information and results should be used when building more robust models with the capacity to predict other relevant quality parameters.

## Figures and Tables

**Table 1:** Calibration and validation data of the chemometric model based on FT-MIR spectroscopy to predict quality parameters in craft beer.

			CALIBRATION (*n* = 45)			VALIDATION (*n* = 15)					
Algorithm	Parameter	Units	Factors	R^2^c	SEC	R^2^v	SEP	MD	RR	%RD	RPD
PCR	Color	EBC	1	0.2403	16.93	-	16.96	-	-	-	-
	Alcohol	%(*v*/*v*)	4	0.7102	1.03	-	1.08	-	-	-	-
	Bitterness	IBU	2	0.4386	16.84	-	16.82	-	-	-	-
	Turbidity	NTU	3	0.3172	16.51	-	16.47	-	-	-	-
	pH	-	1	0.5648	0.24	-	0.25	-	-	-	-
	Acidity	mg/L	2	0.2909	8.38	-	8.72	-	-	-	-
***PLS1***	Color	EBC	10	0.9999	0.06	0.9973	0.07	0.15–0.74	0.59–0.99	2.22–6.57	14.39–18.62
	Alcohol	%(*v*/*v*)	10	0.9999	0.01	0.9890	0.01	0.13–0.68	0.63–1.04	0.76–5.33	13.01–17.93
	Bitterness	IBU	10	0.9999	0.05	0.9948	0.06	0.11–0.79	0.57–1.15	1.61–6.25	9.01–15.32
	Turbidity	NTU	10	0.9999	0.11	0.9964	0.14	0.16–0.68	0.55–1.36	1.35–4.68	11.02–16.97
	pH	-	10	0.9999	0.01	0.9905	0.01	0.09–0.81	0.44–1.28	1.98–4.87	10.48–18.89
	Acidity	mg/L	10	0.9999	0.04	0.9851	0.05	0.18–0.80	0.54–1.45	2.01–6.19	8.32–14.35
PLS2	Color	EBC	5	0.7198	10.64	-	10.95	-	-	-	-
	Alcohol	%(*v*/*v*)	1	0.8604	1.53	-	1.56	-	-	-	-
	Bitterness	IBU	7	0.7371	11.54	-	12.95	-	-	-	-
	Turbidity	NTU	1	0.9240	10.11	-	10.11	-	-	-	-
	pH	-	1	0.7488	0.46	-	0.47	-	-	-	-
	Acidity	mg/L	7	0.7197	8.58	-	8.58	-	-	-	-

The algorithm with the best predictive capacity is given in italics and bold.

**Table 2:** Prediction data of the chemometric model to predict quality parameters in non-craft beer.

Non-Craft Beer	Parameter	Units	MD	RR
1–15	Color	EBC	0.16–0.85	1.95–2.68
	Alcohol	%	0.05–0.62	0.25–1.36
	Bitterness	IBU	0.28–0.37	1.37–1.99
	Turbidity	NTU	0.67–0.89	0.89–2.14
	pH	-	0.31–0.78	0.46–2.38
	Acidity	mg/L	0.47–0.66	1.48–1.69

## Data Availability

The original contributions presented in the study are included in the article/Supplementary Material, further inquiries can be directed to the corresponding author.

## Supplementary Materials

The following supporting information can be downloaded at: https://www.mdpi.com/article/10.3390/foods13081157/s1, Table S1: Color values of craft beer of different styles (*n* = 60); Table S2: Specific gravity values of craft beer of different styles (*n* = 60); Table S3: Alcohol by volume of craft beer of different styles (*n* = 60); Table S4: Bitterness values of craft beer of different styles (*n* = 60); Table S5: Turbidity values of craft beer of different styles (*n* = 60); Table S6: pH and acidity values of craft beer of different styles (*n* = 60); Table S7: Comparison between real value and predicted value of the validation data using Dunnett’s test.
