# Supplementary material for: The Prediction of Quality Parameters of Craft Beer with FT-MIR and Chemometrics
Source: Foods. 2024 Apr 11;13(8):1157. doi: 10.3390/foods13081157 (PMC11049648; doi:10.3390/foods13081157)
Supplement: Supplementary file 1 [file foods-13-01157-s001.zip › foods-2952081-supplementary.pdf]

Table S1: Color values of craft beer of different styles (n = 60).

Beer brands[1]	Beer style[2]	Color (EBC)[3]	EBC (BJCP)[4]	Beer brands[1]	Beer style[2]	Color (EBC)[3]	EBC (BJCP)[4]
1	American Pale Ale	10.1 ± 0.15 $^{p q r}$	5-10	31	India Pale Ale	9.8 ± 0.18 $^{q r s}$	6-14
2	American Pale Ale	5.4 ± 0.03 $^{x y}$	5-10	32	India Pale Ale	8.7 ± 0.03 $^{s t}$	6-14
3	American Pale Ale	5.6 ± 0.14 $^{u v w}$	5-10	33	India Pale Ale	9.3 ± 0.08 $^{r s}$	6-14
4	American Pale Ale	8.4 ± 0.09 $^{s t u}$	5-10	34	India Pale Ale	12.2 ± 0.07 $^{o p}$	6-14
5	American Pale Ale	11.2 ± 0.11 $^{o p}$	5-10	35	India Pale Ale	14.1 ± 0.13 $^{m n}$	6-14
6	American Pale Ale	11.8 ± 0.03 $^{n o p}$	5-10	36	India Pale Ale	7.1 ± 0.02 $^{t u v}$	6-14
7	American Pale Ale	10.4 ± 0.10 $^{m n}$	5-10	37	Kölsch	3.5 ± 0.01 $^{x y z}$	3.5-5
8	American Pale Ale	8.5 ± 0.01 $^{s t u}$	5-10	38	Lager	2.7 ± 0.01 $^{y z}$	2-4
9	Barleywine	18.9 ± 0.09 ^{j}	10-19	39	Lager	2.9 ± 0.01 ^{z}	2-4
10	Belgian Blond Ale	7.1 ± 0.11 $^{s t u}$	4-7	40	Lager	2.2 ± 0.01 ^{z}	2-4
11	Berliner Weisse	2.1 ± 0.03 $^{s t}$	2-3	41	Lager	3.9 ± 0.03 $^{x y z}$	2-4
12	Black India Pale Ale	40.3 ± 0.73 ^{c}	25-40	42	Lager	3.2 ± 0.05 $^{y z}$	2-4
13	Blonde Ale	4.4 ± 0.07 $^{x y}$	3-6	43	Oatmeal Stout	39.7 ± 0.43 ^{c}	22-40
14	Blonde Ale	4.3 ± 0.05 $^{r s}$	3-6	44	Oatmeal Stout	39.4 ± 0.80 ^{a}	22-40
15	Blonde Ale	5.3 ± 0.03 $^{v w x}$	3-6	45	Porter	37.3 ± 0.7 ^{g}	22-40
16	Brown Ale	32.2 ± 0.15 $^{h i}$	18-35	46	Porter	41.3 ± 0.63 ^{e}	22-40
17	Brown Ale	35.5 ± 0.65 ^{g}	18-35	47	Porter	41.1 ± 2.72 ^{e}	22-40
18	Brown Ale	32.3 ± 0.15 $^{h i}$	18-35	48	Porter	40.8 ± 0.20 ^{f}	22-40
19	Brown Ale	31.4 ± 0.05 ^{i}	18-35	49	Porter	39.9 ± 0.20 ^{c}	22-40
20	Brown Ale	30.5 ± 0.04 $^{n o}$	18-35	50	Porter	38.3 ± 1.47 ^{c}	22-40
21	California Common	14.6 ± 0.08 $^{k l}$	10-14	51	Porter	33.8 ± 0.3 ^{h}	22-40
22	Cream Ale	3.5 ± 0.02 $^{x y z}$	2.5-5	52	Scottish Ale	15.6 ± 1.82 $^{l m}$	13-22
23	Cream Ale	3.6 ± 0.04 $^{x y z}$	2.5-5	53	Stout	41.7 ± 1.93 $^{e f}$	30-40
24	Dark Lager	18.1 ± 0.41 $^{j k}$	14-22	54	Stout	39.5 ± 0.56 ^{d}	30-40
25	Dubbel	17.5 ± 0.05 $^{d e}$	10-17	55	Sweet Stout	32.8 ± 0.36 $^{h i}$	30-40
26	Golden Ale	3.8 ± 0.01 $^{x y z}$	2-6	56	Sweet Stout	39.9 ± 0.60 ^{b}	30-40
27	Imperial Stout	39.9 ± 0.62 ^{a}	30-40	57	Vienna	14.3 ± 0.05 ^{j}	9-15
28	Imperial Stout	40.1 ± 0.52 ^{a}	30-40	58	Weissbier	5.7 ± 0.10 $^{o p q}$	2-6
29	India Pale Ale	8.4 ± 0.08 $^{s t u}$	6-14	59	Witbier	3.6 ± 0.04 $^{x y z}$	2-4
30	India Pale Ale	8.1 ± 0.04 $^{s t u}$	6-14	60	Witbier	4.1 ± 0.01 $^{w x y}$	2-4

[1] Beer brands were assigned a number (1-60).
[2] Beer style established by the Beer Judge Certification Program (BJCP).
[3] Color units EBC (European Brewery Convention).
[4] Values accepted by the Beer Judge Certification Program (1-65 EBC).
Values represent means ± standard deviation. Means with different letters per column indicate significant statistical differences (Tukey, $p \leq 0.05$).

Table S2: Specific gravity values of craft beer of different styles (n = 60).

Beer brands[1]	Beer style[2]	SG[3]	SG (BJCP)[4]	Beer brands[1]	Beer style[2]	SG[3]	SG (BJCP)[4]
1	American Pale Ale	$1.013 \pm 0.01^{\text{z aa ab}}$	1.010-1.015	31	India Pale Ale	$1.011 \pm 0.06^{\text{v w x y z aa}}$	1.008-1.014
2	American Pale Ale	$1.016 \pm 0.01^{\text{t u v w x y z}}$	1.010-1.015	32	India Pale Ale	$1.012 \pm 0.05^{\text{n o p q r s t u}}$	1.008-1.014
3	American Pale Ale	$1.012 \pm 0.01^{\text{j k l m n}}$	1.010-1.015	33	India Pale Ale	$1.011 \pm 0.08^{\text{s t u v w x y}}$	1.008-1.014
4	American Pale Ale	$1.012 \pm 0.02^{\text{j k l m n o p}}$	1.010-1.015	34	India Pale Ale	$1.014 \pm 0.04^{\text{u v w x y z aa}}$	1.008-1.014
5	American Pale Ale	$1.010 \pm 0.01^{\text{o p q r s t u}}$	1.010-1.015	35	India Pale Ale	$1.014 \pm 0.06^{\text{h i j k}}$	1.008-1.014
6	American Pale Ale	$1.010 \pm 0.03^{\text{l m n o p q r s}}$	1.010-1.015	36	India Pale Ale	$1.008 \pm 0.02^{\text{q r s t u v w}}$	1.008-1.014
7	American Pale Ale	$1.015 \pm 0.02^{\text{t u v w x y z}}$	1.010-1.015	37	Kölsch	$1.011 \pm 0.02^{\text{k l m n o p}}$	1.007-1.011
8	American Pale Ale	$1.015 \pm 0.01^{\text{r s t u v w x}}$	1.010-1.015	38	Lager	$1.005 \pm 0.04^{\text{w x y z aa ab}}$	1.004-1.010
9	Barleywine	$1.019 \pm 0.02^{\text{c d e}}$	1.016-1.030	39	Lager	$1.010 \pm 0.06^{\text{i j k l}}$	1.004-1.010
10	Belgian Blond Ale	$1.008 \pm 0.02^{\text{y z aa ab}}$	1.008-1.018	40	Lager	$1.008 \pm 0.07^{\text{p q r s t u v}}$	1.004-1.010
11	Berliner Weisse	$1.006 \pm 0.01^{\text{j k l m}}$	1.003-1.006	41	Lager	$1.007 \pm 0.04^{\text{t u v w x y z}}$	1.004-1.010
12	Black India Pale Ale	$1.006 \pm 0.04^{\text{t u v w x y z}}$	1.010-1.018	42	Lager	$1.004 \pm 0.08^{\text{aa ab}}$	1.004-1.010
13	Blonde Ale	$1.015 \pm 0.04^{\text{f g h i}}$	1.008-1.018	43	Oatmeal Stout	$1.018 \pm 0.09^{\text{c d}}$	1.010-1.018
14	Blonde Ale	$1.008 \pm 0.05^{\text{o p r s t u v}}$	1.008-1.018	44	Oatmeal Stout	$1.020 \pm 0.03^{\text{a}}$	1.010-1.018
15	Blonde Ale	$1.018 \pm 0.02^{\text{d e f}}$	1.008-1.018	45	Porter	$1.009 \pm 0.07^{\text{u v w x y z aa}}$	1.008-1.014
16	Brown Ale	$1.013 \pm 0.01^{\text{u v w x y z aa}}$	1.008-1.013	46	Porter	$1.014 \pm 0.08^{\text{e f g h}}$	1.008-1.014
17	Brown Ale	$1.013 \pm 0.05^{\text{g h i j}}$	1.008-1.013	47	Porter	$1.014 \pm 0.06^{\text{c d}}$	1.008-1.014
18	Brown Ale	$1.009 \pm 0.01^{\text{m n o p q r s t}}$	1.008-1.013	48	Porter	$1.014 \pm 0.09^{\text{g h i j}}$	1.008-1.014
19	Brown Ale	$1.008 \pm 0.04^{\text{w x y z aa ab}}$	1.008-1.013	49	Porter	$1.013 \pm 0.04^{\text{i j k l}}$	1.008-1.014
20	Brown Ale	$1.013 \pm 0.05^{\text{h i j k}}$	1.008-1.013	50	Porter	$1.011 \pm 0.06^{\text{j k l m n o p}}$	1.008-1.014
21	California Common	$1.014 \pm 0.04^{\text{c}}$	1.011-1.014	51	Porter	$1.010 \pm 0.08^{\text{d e f}}$	1.008-1.014
22	Cream Ale	$1.007 \pm 0.03^{\text{x y z aa ab}}$	1.006-1.012	52	Scottish Ale	$1.013 \pm 0.03^{\text{i j k l}}$	1.010-1.016
23	Cream Ale	$1.010 \pm 0.02^{\text{g h i j}}$	1.006-1.012	53	Stout	$1.012 \pm 0.02^{\text{q r s t u v w}}$	1.010-1.022
24	Dark Lager	$1.012 \pm 0.06^{\text{e f g}}$	1.008-1.012	54	Stout	$1.010 \pm 0.04^{\text{l m n o p q r}}$	1.010-1.022
25	Dubbel	$1.018 \pm 0.03^{\text{i j k l}}$	1.008-1.018	55	Sweet Stout	$1.014 \pm 0.05^{\text{h i j k}}$	1.012-1.024
26	Golden Ale	$1.006 \pm 0.07^{\text{ab}}$	1.006-1.012	56	Sweet Stout	$1.012 \pm 0.04^{\text{j k l m n o}}$	1.012-1.024
27	Imperial Stout	$1.018 \pm 0.06^{\text{i j k l}}$	1.018-1.030	57	Vienna	$1.010 \pm 0.03^{\text{k l m n o p q}}$	1.010-1.014
28	Imperial Stout	$1.030 \pm 0.03^{\text{b}}$	1.018-1.030	58	Weissbier	$1.013 \pm 0.06^{\text{t u v w x y z}}$	1.010-1.014
29	India Pale Ale	$1.008 \pm 0.05^{\text{z aa ab}}$	1.008-1.014	59	Witbier	$1.008 \pm 0.04^{\text{x y z aa ab}}$	1.008-1.012
30	India Pale Ale	$1.012 \pm 0.08^{\text{i j k l}}$	1.008-1.014	60	Witbier	$1.010 \pm 0.02^{\text{u v w x y z aa}}$	1.008-1.012

[1] Beer brands were assigned a number (1-60).
[2] Beer style established by the Beer Judge Certification Program (BJCP).
[3] SG (Specific Gravity).
[4] Values accepted by the Beer Judge Certification Program (1.001-1.040).
Values represent means ± standard deviation. Means with different letters per column indicate significant statistical differences (Tukey, $p \leq 0.05$).

Table S3: Alcohol by volume of craft beer of different styles (n = 60).

Beer brands[1]	Beer style[2]	Alcohol (%, v/v)[3]	Alcohol on label (%, v/v)[4]	Beer brands[1]	Beer style[2]	Alcohol (%, v/v)[3]	Alcohol on label (%, v/v)[4]
1	American Pale Ale	4.7 ± 0.47 $^{ab\ ac\ ad}$	5	31	India Pale Ale	7.1 ± 0.57 $^{h\ i\ j}$	7
2	American Pale Ale	6.0 ± 0.47 $^{f\ g}$	5	32	India Pale Ale	5.9 ± 0.00 $^{f\ g\ h}$	5.7
3	American Pale Ale	6.2 ± 0.0 $^{c\ d}$	6.5	33	India Pale Ale	7.2 ± 0.00 $^{k\ l\ m\ n\ o\ p}$	7.3
4	American Pale Ale	5.1 ± 0.47 $^{z\ aa\ ab}$	5.2	34	India Pale Ale	5.9 ± 0.00 $^{m\ n\ o\ p\ q}$	5.8
5	American Pale Ale	4.7 ± 0.00 $^{u\ v\ w\ x\ y\ z}$	4.5	35	India Pale Ale	6.8 ± 0.00 $^{i\ j\ k\ l}$	6.4
6	American Pale Ale	5.6 ± 0.00 $^{i\ j\ k\ l\ m}$	6	36	India Pale Ale	7.1 ± 0.47 $^{e\ f}$	7
7	American Pale Ale	5.6 ± 0.00 $^{i\ j\ k\ l\ m}$	5.9	37	Kölsch	5.6 ± 0.00 $^{w\ x\ y\ z}$	5
8	American Pale Ale	5.1 ± 0.00 $^{l\ m\ n\ o\ p}$	5.2	38	Lager	4.9 ± 0.00 $^{z\ aa\ ab}$	4.8
9	Barleywine	10.4 ± 0.00 ^{a}	10	39	Lager	4.5 ± 0.00 ^{ad}	3.9
10	Belgian Blond Ale	6.3 ± 0.00 $^{f\ g\ h\ i}$	6.2	40	Lager	5.1 ± 0.00 $^{ac\ ad}$	4.2
11	Berliner Weisse	4.0 ± 0.00 $^{s\ t\ u\ v\ w\ x\ y}$	3.5	41	Lager	5.4 ± 0.00 $^{p\ q\ r\ s\ t\ u\ v}$	5
12	Black India Pale Ale	6.2 ± 0.00 $^{g\ h\ i}$	6.5	42	Lager	4.8 ± 0.00 $^{aa\ ab\ ac}$	4
13	Blonde Ale	4.6 ± 0.00 $^{x\ y\ z\ aa}$	4.5	43	Oatmeal Stout	4.5 ± 0.00 $^{t\ u\ v\ w\ x\ y}$	4.2
14	Blonde Ale	4.6 ± 0.00 $^{o\ p\ q\ r\ s\ t}$	5	44	Oatmeal Stout	10.0 ± 0.00 ^{b}	10
15	Blonde Ale	5.3 ± 0.00 $^{y\ z\ aa\ ab}$	5	45	Porter	6.5 ± 0.00 $^{s\ t\ u\ v\ w\ x\ y}$	6
16	Brown Ale	6.4 ± 0.00 $^{p\ q\ r\ s\ t\ u\ v\ w}$	6	46	Porter	6.5 ± 0.47 $^{h\ i\ j}$	6.2
17	Brown Ale	4.6 ± 0.47 $^{w\ x\ y\ z}$	4.8	47	Porter	6.3 ± 0.47 $^{i\ j\ k}$	6.2
18	Brown Ale	5.3 ± 0.47 $^{f\ g\ h\ i}$	5	48	Porter	5.0 ± 0.00 $^{n\ o\ p\ r\ s}$	5.5
19	Brown Ale	5.9 ± 0.00 $^{i\ j\ k}$	5.5	49	Porter	5.5 ± 0.00 $^{p\ q\ r\ s\ t}$	5.5
20	Brown Ale	5.1 ± 0.00 $^{q\ r\ s\ t\ u\ v\ w\ x}$	5.3	50	Porter	5.8 ± 0.47 $^{d\ e}$	5.2
21	California Common	5.5 ± 0.00 $^{x\ y\ z\ aa}$	5	51	Porter	4.6 ± 0.00 $^{o\ p\ q\ r\ s\ t}$	4.6
22	Cream Ale	5.5 ± 0.00 $^{p\ q\ r\ s\ t}$	5	52	Scottish Ale	6.4 ± 0.00 $^{p\ q\ r\ s\ t\ u\ v}$	6.1
23	Cream Ale	4.3 ± 0.47 $^{y\ z\ aa\ ab}$	4.5	53	Stout	4.8 ± 0.00 $^{m\ n\ o\ p\ q\ r}$	5
24	Dark Lager	5.5 ± 0.00 $^{p\ q\ r\ s\ t\ u}$	5	54	Stout	5.6 ± 0.00 $^{i\ j\ k\ l\ m\ n}$	5.5
25	Dubbel	6.6 ± 0.00 $^{b\ c}$	6.4	55	Sweet Stout	9.2 ± 0.00 ^{b}	9
26	Golden Ale	5.1 ± 0.47 $^{q\ r\ s\ t\ u\ v\ w\ x}$	5	56	Sweet Stout	6.0 ± 0.00 $^{m\ n\ o\ p}$	6
27	Imperial Stout	9.0 ± 0.00 $^{f\ g}$	9	57	Vienna	5.5 ± 0.47 $^{v\ w\ x\ y\ z}$	5
28	Imperial Stout	8.1 ± 0.00 $^{i\ j}$	8	58	Weissbier	4.4 ± 0.00 $^{p\ q\ r\ s\ t\ u\ v}$	4.8
29	India Pale Ale	7.8 ± 0.00 $^{n\ o\ p\ q\ r\ s}$	7	59	Witbier	5.0 ± 0.00 $^{j\ k\ l\ m\ n\ o}$	4.8
30	India Pale Ale	7.3 ± 0.00 $^{f\ g\ h\ i}$	7.1	60	Witbier	5.0 ± 0.00 $^{r\ s\ t\ u\ v\ w\ x\ y}$	5

[1] Beer brands were assigned a number (1-60).
[2] Beer style established by the Beer Judge Certification Program (BJCP).
[3] Alcohol (%, v/v).
[4] Alcohol (%, v/v) reported by the manufacturer.
Values accepted by NOM-199-SCFI-2017 (> 2% < 20%, v/v).
Values represent means ± standard deviation. Means with different letters per column indicate significant statistical differences (Tukey, $p \leq 0.05$).

Table S4: Bitterness values of craft beer of different styles (n = 60).

Beer brands[1]	Beer style[2]	Bitterness (IBU)[3]	IBU (BJCP)[4]	Beer brands[1]	Beer style[2]	Bitterness (IBU)[3]	IBU (BJCP)[4]
1	American Pale Ale	33.1 ± 1.05 $^{\text{g h i j}}$	30-50	31	India Pale Ale	47.5 ± 5.5 ^{d}	40-70
2	American Pale Ale	34.5 ± 0.85 $^{\text{m n o p}}$	30-50	32	India Pale Ale	39.0 ± 2.81 $^{\text{f g}}$	40-70
3	American Pale Ale	38.0 ± 0.16 $^{\text{j k l m}}$	30-50	33	India Pale Ale	66.0 ± 1.72 $^{\text{b c}}$	40-70
4	American Pale Ale	41.1 ± 1.37 $^{\text{e f}}$	30- 50	34	India Pale Ale	65.0 ± 1.57 $^{\text{b c}}$	40-70
5	American Pale Ale	31.0 ± 1.83 $^{\text{o p q r s t}}$	30-50	35	India Pale Ale	39.0 ± 1.48 $^{\text{f g}}$	40-70
6	American Pale Ale	42.1 ± 1.07 $^{\text{w x y z aa ab ac ad}}$	30-50	36	India Pale Ale	63.0 ± 0.87 ^{c}	40-70
7	American Pale Ale	36.0 ± 0.92 $^{\text{f g h i}}$	30-50	37	Kölsch	19.0 ± 0.65 $^{\text{v w x y z aa ab ac}}$	18-30
8	American Pale Ale	46.1 ± 0.46 $^{\text{s t u v w x y}}$	30-50	38	Lager	15.5 ± 2.18 $^{\text{f g h i}}$	8-18
9	Barleywine	55.5 ± 1.96 $^{\text{d e}}$	50-100	39	Lager	17.0 ± 0.72 $^{\text{r s t u v w}}$	8-18
10	Belgian Blond Ale	23.0 ± 2.62 $^{\text{m n o p q r}}$	15-30	40	Lager	16.5 ± 1.27 $^{\text{k l m n o}}$	8-18
11	Berliner Weisse	14.5 ± 0.40 $^{\text{u v wx y z aa}}$	3-8	41	Lager	9.0 ± 1.16 $^{\text{aa ab ac ad ae}}$	8-18
12	Black India Pale Ale	70.0 ± 1.26 ^{b}	50-90	42	Lager	14.0 ± 0.25 ^{ae}	8-18
13	Blonde Ale	15.0 ± 0.30 $^{\text{z aa ab ac ad ae}}$	15-28	43	Oatmeal Stout	38.5 ± 1.33 $^{\text{aa ab ac ad ae}}$	25-40
14	Blonde Ale	15.5 ± 1.42 $^{\text{y z aa ab ac ad}}$	15-28	44	Oatmeal Stout	29.0 ± 1.67 $^{\text{j k l m}}$	25-40
15	Blonde Ale	15.5 ± 1.12 $^{\text{w x y z aa ab ac ad}}$	15-28	45	Porter	29.5 ± 1.22 $^{\text{x y z aa ab ac ad}}$	25-50
16	Brown Ale	20.5 ± 0.55 $^{\text{v w x y z aa ab ac}}$	20-30	46	Porter	27.5 ± 1.08 $^{\text{j k l m n}}$	25-50
17	Brown Ale	22.0 ± 0.48 $^{\text{n o p q r s}}$	20-30	47	Porter	23.5 ± 0.65 $^{\text{m n o p q}}$	25-50
18	Brown Ale	30.5 ± 2.31 $^{\text{e f}}$	20-30	48	Porter	28.0 ± 0.72 $^{\text{j k l m}}$	25-50
19	Brown Ale	33.0 ± 3.9 $^{\text{d e}}$	20-30	49	Porter	27.5 ± 0.40 $^{\text{j k l m n}}$	25-50
20	Brown Ale	31.0 ± 0.68 $^{\text{i j k l}}$	20-30	50	Porter	25.0 ± 0.58 $^{\text{l m n o p}}$	25-50
21	California Common	32.0 ± 2.47 $^{\text{m n o p q r}}$	30-45	51	Porter	26.5 ± 1.24 $^{\text{s t u v w x}}$	25-50
22	Cream Ale	18.5 ± 2.05 $^{\text{q r s t u v}}$	8-20	52	Scottish Ale	16.5 ± 0.46 $^{\text{ad ae}}$	15-30
23	Cream Ale	14.0 ± 2.49 $^{\text{u v w x y z aa ab}}$	8-20	53	Stout	39.0 ± 1.31 $^{\text{j k l m}}$	35-75
24	Dark Lager	21.0 ± 0.35 $^{\text{l m n o p}}$	8-20	54	Stout	38.1 ± 1.92 $^{\text{f g h}}$	35-75
25	Dubbel	18.5 ± 2.00 $^{\text{q r s t u v}}$	15-25	55	Sweet Stout	32.0 ± 0.30 $^{\text{i j k}}$	20-40
26	Golden Ale	29.0 ± 2.35 $^{\text{j k l m}}$	20-45	56	Sweet Stout	28.0 ± 1.49 $^{\text{w x y z aa ab ac ad}}$	20-40
27	Imperial Stout	64.5 ± 4.40 $^{\text{b c}}$	50-90	57	Vienna	25.5 ± 1.42 $^{\text{t u v w x y z}}$	18-30
28	Imperial Stout	72.0 ± 0.86 $^{\text{h i j k}}$	50-90	58	Weissbier	11.0 ± 0.62 $^{\text{x y z aa ab ac ad}}$	8-15
29	India Pale Ale	65.5 ± 2.70 $^{\text{b c}}$	40-70	59	Witbier	17.0 ± 1.72 $^{\text{ac ad ae}}$	8-20
30	India Pale Ale	69.0 ± 0.81 ^{a}	40-70	60	Witbier	19.5 ± 1.34 $^{\text{p q r s t u}}$	8-20

[1] Beer brands were assigned a number (1-60).
[2] Beer style established by the Beer Judge Certification Program (BJCP).
[3] IBU (International Bitterness Units).
[4] Values accepted by the Beer Judge Certification Program (1-120 IBU).
Values represent means ± standard deviation. Means with different letters per column indicate significant statistical differences (Tukey, p ≤ 0.05).

Table S5: Turbidity values of craft beer of different styles (n = 60).

Beer brands[1]	Beer style[2]	Turbidity (NTU)[3]	Beer brands[1]	Beer style[2]	Turbidity (NTU)[3]
1	American Pale Ale	35.3 ± 1.15 ^{h}	31	India Pale Ale	22.3 ± 2.08 k ^{l}
2	American Pale Ale	5.0 ± 0.00 $^{ab\ ac\ ad\ ae\ af}$	32	India Pale Ale	3.0 ± 0.00 $^{af\ ag}$
3	American Pale Ale	38.3 ± 0.57 ^{g}	33	India Pale Ale	13.0 ± 1.00 $^{r\ s\ t}$
4	American Pale Ale	4.0 ± 1.00 $^{ad\ ae\ af\ ag}$	34	India Pale Ale	7.0 ± 0.00 $^{x\ y\ z\ aa\ ab\ ac}$
5	American Pale Ale	10.0 ± 0.00 $^{u\ v\ w}$	35	India Pale Ale	21.0 ± 0.00 $^{k\ l\ m\ n}$
6	American Pale Ale	5.0 ± 0.00 $^{ab\ ac\ ad\ ae\ af}$	36	India Pale Ale	3.3 ± 0.57 $^{ae\ af\ ag}$
7	American Pale Ale	6.0 ± 0.00 $^{y\ z\ aa\ ab\ ac\ ad}$	37	Kölsch	19.3 ± 1.52 $^{m\ n}$
8	American Pale Ale	6.6 ± 0.57 $^{x\ y\ z\ aa\ ab\ ac}$	38	Lager	3.0 ± 1.00 $^{af\ ag}$
9	Barleywine	11.0 ± 0.00 $^{t\ u\ v}$	39	Lager	2.0 ± 0.00 ^{ag}
10	Belgian Blond Ale	8.0 ± 0.00 $^{w\ x\ y\ z}$	40	Lager	5.0 ± 1.00 $^{ab\ ac\ ad\ ae\ af}$
11	Berliner Weisse	20.0 ± 0.00 $^{l\ m\ n}$	41	Lager	11.3 ± 0.57 $^{s\ t\ u\ v}$
12	Black India Pale Ale	25.3 ± 1.15 $^{i\ j}$	42	Lager	8.3 ± 0.57 $^{w\ x\ y}$
13	Blonde Ale	15.3 ± 0.57 $^{p\ q\ r}$	43	Oatmeal Stout	13.6 ± 0.57 $^{q\ r\ s}$
14	Blonde Ale	22.0 ± 0.00 $^{k\ l}$	44	Oatmeal Stout	114.3 ± 0.57 ^{b}
15	Blonde Ale	5.3 ± 0.57 $^{aa\ ab\ ac\ ad\ ae\ af}$	45	Porter	16.0 ± 1.00 $^{p\ q}$
16	Brown Ale	4.6 ± 0.57 $^{ac\ ad\ ae\ af}$	46	Porter	16.6 ± 0.57 $^{o\ p}$
17	Brown Ale	7.3 ± 0.57 $^{x\ y\ z\ aa}$	47	Porter	13.0 ± 0.00 $^{r\ s\ t}$
18	Brown Ale	5.6 ± 0.57 $^{z\ aa\ ab\ ac\ ad\ ae}$	48	Porter	56.3 ± 2.08 ^{d}
19	Brown Ale	19.0 ± 1.00 $^{n\ o}$	49	Porter	12.6 ± 0.57 $^{s\ t}$
20	Brown Ale	21.6 ± 1.52 $^{k\ l\ m}$	50	Porter	4.0 ± 0.00 $^{ad\ ae\ af\ ag}$
21	California Common	39.6 ± 0.57 ^{g}	51	Porter	47.0 ± 0.57 ^{e}
22	Cream Ale	39.3 ± 0.57 ^{g}	52	Scottish Ale	9.0 ± 0.00 $^{v\ w\ x}$
23	Cream Ale	8.0 ± 0.00 $^{w\ x\ y\ z}$	53	Stout	25.6 ± 0.57 ^{i}
24	Dark Lager	44.3 ± 0.57 ^{f}	54	Stout	7.3 ± 0.57 $^{x\ y\ z\ aa\ ab}$
25	Dubbel	7.0 ± 0.57 $^{w\ x\ y\ z\ aa}$	55	Sweet Stout	23.0 ± 0.00 $^{j\ k}$
26	Golden Ale	9.0 ± 0.00 $^{v\ w\ x}$	56	Sweet Stout	64.0 ± 0.00 ^{c}
27	Imperial Stout	12.0 ± 1.00 $^{s\ t\ u}$	57	Vienna	6.3 ± 1.15 $^{y\ z\ aa\ ab\ ac\ ad}$
28	Imperial Stout	201.3 ± 1.52 ^{a}	58	Weissbier	26.0 ± 0.00 ^{i}
29	India Pale Ale	10.0 ± 0.00 $^{u\ v\ w}$	59	Witbier	16.3 ± 0.57 ^{p}
30	India Pale Ale	10.0 ± 1.00 $^{u\ v\ w}$	60	Witbier	11.0 ± 0.00 $^{t\ u\ v}$

[1] Beer brands were assigned a number (1-60).
[2] Beer style established by the Beer Judge Certification Program (BJCP).
[3] NTU (Nephelometric Turbidity Units).
Values represent means ± standard deviation. Means with different letters per column indicate significant statistical differences (Tukey, $p \leq 0.05$).

Table S6: pH and acidity values of craft beer of different styles (n = 60).

Beer brands[1]	Beer style[2]	pH	Acidity (mg/L)[3]	Beer brands[1]	Beer style[2]	pH	Acidity (mg/L)[3]
1	American Pale Ale	4.06 ± 0.01 [u v w x]	20.76 ± 1.36 [j k l m n]	31	India Pale Ale	4.30 ± 0.01 [l m n o]	20.16 ± 1.56 [k l m n o]
2	American Pale Ale	3.92 ± 0.03 [y z aa]	18.60 ± 0.74 [l m n o p q]	32	India Pale Ale	4.61 ± 0.01 [c d e]	25.44 ± 1.81 [h i]
3	American Pale Ale	4.21 ± 0.02 [o p q r s]	22.08 ± 0.54 [i j k l]	33	India Pale Ale	4.63 ± 0.00 [c d]	20.88 ± 0.72 [j k l m]
4	American Pale Ale	4.23 ± 0.00 [o p q r s]	19.08 ± 1.29 [l m n o p]	34	India Pale Ale	4.54 ± 0.01 [d e f g]	12.72 ± 0.41 [s t u v w]
5	American Pale Ale	3.85 ± 0.01 [aa ab]	19.08 ± 1.08 [l m n o p]	35	India Pale Ale	3.77 ± 0.00 [ab ac ad]	18.72 ± 0.36 [l m n o p r]
6	American Pale Ale	4.23 ± 0.03 [o p q r]	18.84 ± 1.36 [l m n o p]	36	India Pale Ale	4.42 ± 0.02 [h i j k]	17.88 ± 0.41 [m n o p r]
7	American Pale Ale	4.51 ± 0.00 [e f g h]	10.44 ± 0.95 [v w x y]	37	Kölsch	4.52 ± 0.05 [e f g]	10.92 ± 1.15 [u v w x]
8	American Pale Ale	4.30 ± 0.05 [l m n o]	17.52 ± 0.74 [n o p q r]	38	Lager	3.90 ± 0.05 [z aa]	12.84 ± 0.54 [s t u v w]
9	Barleywine	4.70 ± 0.03 [b c]	24.24 ± 1.09 [h i j]	39	Lager	3.84 ± 0.01 [aa ab ac]	10.8 ± 0.95 [u v w x]
10	Belgian Blond Ale	4.29 ± 0.03 [l m n o]	26.52 ± 0.90 [h]	40	Lager	4.23 ± 0.01 [o p r s]	6.36 ± 2.16 [z aa]
11	Berliner Weisse	3.46 ± 0.03 [ae]	32.16 ± 0.90 [d e]	41	Lager	3.69 ± 0.03 [ad]	18.72 ± 0.72 [l m n o p q]
12	Black India Pale Ale	4.36 ± 0.03 [j k l m]	26.76 ± 2.16 [g h]	42	Lager	4.15 ± 0.06 [q r s t u]	14.76 ± 0.72 [q r s t u]
13	Blonde Ale	3.51 ± 0.00 [ae]	19.92 ± 0.41 [l m n o p]	43	Oatmeal Stout	4.15 ± 0.01 [q r s t u]	17.52 ± 1.45 [n o p q r]
14	Blonde Ale	4.11 ± 0.02 [s t u v w]	18.96 ± 1.98 [l m n o p]	44	Oatmeal Stout	5.03 ± 0.02 [a]	47.76 ± 0.54 [a]
15	Blonde Ale	4.16 ± 0.02 [p q r s t u]	4.92 ± 1.36 [aa]	45	Porter	3.53 ± 0.01 [ae]	38.04 ± 1.36 [b c]
16	Brown Ale	4.27 ± 0.03 [m n o p]	14.4 ± 0.95 [r s t u v]	46	Porter	3.91 ± 0.00 [z aa]	16.56 ± 0.36 [o p q r s]
17	Brown Ale	3.96 ± 0.03 [x y z]	12.36 ± 1.45 [t u v w]	47	Porter	4.08 ± 0.01 [t u v w]	27.00 ± 1.24 [g h]
18	Brown Ale	4.57 ± 0.01 [d e f]	31.80 ± 1.26 [d e f]	48	Porter	4.27 ± 0.01 [m n o p]	32.16 ± 0.54 [d e]
19	Brown Ale	4.38 ± 0.01 [i j k l]	12.72 ± 2.39 [s t u v w]	49	Porter	4.08 ± 0.00 [t u v w]	27.36 ± 1.44 [g h]
20	Brown Ale	3.74 ± 0.08 [ac ad]	16.44 ± 0.20 [o p q r s]	50	Porter	4.17 ± 0.00 [p q r s t]	18.72 ± 1.24 [k l m n o]
21	California Common	4.29 ± 0.01 [l m n o]	16.44 ± 0.20 [o p q r s]	51	Porter	4.02 ± 0.00 [w x y]	9.48 ± 1.70 [w x y z]
22	Cream Ale	3.84 ± 0.06 [aa ab ac]	27.84 ± 0.74 [f g h]	52	Scottish Ale	4.24 ± 0.01 [n o p q]	18.24 ± 0.54 [l m n o p q r]
23	Cream Ale	4.14 ± 0.01 [q r s t u]	16.44 ± 0.74 [o p q r s]	53	Stout	4.34 ± 0.00 [k l m n]	16.08 ± 1.15 [p q r s t]
24	Dark Lager	4.13 ± 0.02 [r s t u v]	17.76 ± 1.09 [n o p q r s]	54	Stout	4.27 ± 0.02 [m n o p]	27.72 ± 1.08 [g h]
25	Dubbel	3.92 ± 0.01 [y z aa]	40.68 ± 0.95 [b]	55	Sweet Stout	4.23 ± 0.00 [o p q r]	27.36 ± 1.90 [g h]
26	Golden Ale	4.36 ± 0.01 [j k l m n]	8.04 ± 1.45 [x y z aa]	56	Sweet Stout	4.03 ± 0.02 [v w x]	19.80 ± 0.0 [l m n o p]
27	Imperial Stout	4.45 ± 0.03 [g h i j]	37.92 ± 1.98 [b c]	57	Vienna	4.21 ± 0.01 [o p q r s]	6.72 ± 1.84 [y z aa]
28	Imperial Stout	4.75 ± 0.00 [b]	40.32 ± 0.62 [b]	58	Weissbier	4.06 ± 0.03 [u v w x]	20.64 ± 0.74 [j k l m n]
29	India Pale Ale	4.47 ± 0.05 [f g h i]	21.84 ± 0.74 [i j k l m]	59	Witbier	4.49 ± 0.04 [f g h]	30.72 ± 0.20 [e f g]
30	India Pale Ale	4.76 ± 0.05 [b]	24.12 ± 0.36 [h i j k]	60	Witbier	3.35 ± 0.05 [af]	35.40 ± 0.54 [c d]

[1] Beer brands were assigned a number (1-60).
[2] Beer style established by the Beer Judge Certification Program (BJCP).
[3] Values accepted by NOM-199-SCFI-2007: ≤ 10 000 mg lactic acid/L.
Values represent means ± standard deviation. Means with different letters per column indicate significant statistical differences (Tukey, $p \leq 0.05$).

Table S7: Comparison between real value and predicted value of the validation data using a Dunnett's test.

**Sample**	**Parameter**	**Real value**	**Predicted value**	**Grouping**	**Sample**	**Parameter**	**Real value**	**Predicted value**	**Grouping**	**Sample**	**Parameter**	**Real value**	**Predicted value**	**Grouping**
1	Color	32.20	33.57	A	6	Color	8.70	7.94	A	11	Color	14.40	14.58	A
	Alcohol	4.40	4.49	A		Alcohol	6.90	6.76	A		Alcohol	5.60	5.46	A
	Bitterness	12.50	12.02	A		Bitterness	39.00	40.88	A		Bitterness	36.00	35.23	A
	Turbidity	4.60	4.35	A		Turbidity	3.00	3.45	A		Turbidity	6.00	5.42	A
	pH	4.27	4.24	A		pH	4.61	4.62	A		pH	4.51	4.50	A
	Acidity	14.40	14.28	A		Acidity	25.44	25.11	A		Acidity	10.44	10.73	A
2	Color	4.40	4.41	A	7	Color	12.20	11.67	A	12	Color	9.10	8.22	A
	Alcohol	7.00	6.89	A		Alcohol	3.70	3.95	A		Alcohol	4.00	3.97	A
	Bitterness	24.50	23.76	A		Bitterness	21.00	20.62	A		Bitterness	14.50	14.27	A
	Turbidity	5.00	5.51	A		Turbidity	10.00	10.76	A		Turbidity	20.00	19.49	A
	pH	3.92	3.91	A		pH	3.85	3.83	A		pH	3.46	3.48	A
	Acidity	18.60	19.63	A		Acidity	19.08	19.13	A		Acidity	32.16	31.01	A
3	Color	8.40	8.27	A	8	Color	4.40	4.58	A	13	Color	48.30	47.33	A
	Alcohol	3.10	2.99	A		Alcohol	3.60	3.76	A		Alcohol	4.50	4.69	A
	Bitterness	41.00	40.20	A		Bitterness	10.00	10.46	A		Bitterness	25.00	24.73	A
	Turbidity	4.23	4.29	A		Turbidity	15.30	14.84	A		Turbidity	4.00	4.17	A
	pH	19.08	19.70	A		pH	3.51	3.54	A		pH	4.17	4.17	A
	Acidity	4.00	4.52	A		Acidity	18.92	18.56	A		Acidity	18.72	19.26	A
4	Color	2.20	2.20	A	9	Color	9.30	10.06	A	14	Color	16.16	16.67	A
	Alcohol	2.10	2.45	A		Alcohol	4.60	4.85	A		Alcohol	3.50	3.81	A
	Bitterness	26.50	27.50	A		Bitterness	10.50	10.90	A		Bitterness	23.00	24.43	A
	Turbidity	5.00	5.29	A		Turbidity	22.00	21.03	A		Turbidity	39.60	40.75	A
	pH	4.23	4.26	A		pH	4.11	4.14	A		pH	4.29	4.23	A
	Acidity	6.36	7.29	A		Acidity	19.96	19.71	A		Acidity	16.44	15.89	A
5	Color	9.80	9.64	A	10	Color	56.00	55.61	A	15	Color	18.00	17.08	A
	Alcohol	6.10	6.00	A		Alcohol	5.00	5.06	A		Alcohol	4.50	4.40	A
	Bitterness	47.50	47.23	A		Bitterness	12.00	12.46	A		Bitterness	25.00	24.11	A
	Turbidity	22.30	23.13	A		Turbidity	23.00	22.72	A		Turbidity	44.30	43.94	A
	pH	4.30	4.34	A		pH	4.03	4.06	A		pH	4.13	4.11	A
	Acidity	20.16	19.75	A		Acidity	19.80	19.52	A		Acidity	17.76	16.61	A

Different letters per row indicate significant statistical differences ($p \leq 0.05$) for each parameter per sample.
